# Linkage Between Inclusive Digital Finance and High-Tech Enterprise Innovation Performance: Role of Debt and Equity Financing

**DOI:** 10.3389/fpsyg.2021.814408

**Published:** 2021-12-28

**Authors:** Huiyuan Han, Xiaomin Gu

**Affiliations:** ^1^Glorious Sun School of Business Management, Donghua University, Shanghai, China; ^2^Financial Technology College, Shanghai Lixin University of Accounting and Finance, Shanghai, China

**Keywords:** inclusive digital finance, external financing, innovation performance, digital economy, sustainability, information communication technology, equity financing

## Abstract

This study investigates the relationship between digital financial inclusion, external financing, and the innovation performance of high-tech enterprises in China. The choice of corporate financing methods is an important part of organizational behavioral psychology, and different financing models will have a certain effect on organizational performance, especially in the digital economy environment. Therefore, based on resource dependence theory and financing constraint theory, the present study utilizes the panel data collected from the China Stock Market & Accounting Research (CSMAR) database from 2011 to 2020 of 112 companies in the Yangtze River Delta region and the “The Peking University Digital Financial Inclusion Index of China (PKU-DFIIC)” released by the Peking University Digital Finance Research Center and Ant Financial Group. The results show that the Digital Financial Inclusion Index (DFIIC) has a significant positive correlation with the innovation performance of high-tech enterprises. The higher the level of debt financing, the stronger the role of digital financial inclusion in promoting innovation performance. Investigating the DFIIC in terms of coverage breadth and usage depth, we find that usage depth does not significantly encourage innovation performance. The effect of the interaction between coverage breadth and external financing is consistent with the results for the DFIIC. The study suggests that equity financing promotes the usage depth of the DFIIC in state-owned enterprises. In contrast, debt financing promotes the coverage breadth of non-state-owned enterprises. Finally, we propose relevant policy recommendations based on the research results. It includes in-depth popularization of inclusive finance in the daily operations of enterprises at the technical level, refinement of external financing policy incentives for enterprises based on the characteristics of ownership, and strengthening the research of technologies such as big data, artificial intelligence (AI), and cloud computing. The paper presents a range of theoretical and practical implications for practitioners and academics relevant to high-tech enterprises.

## Introduction

The digital economy is developing rapidly, and the need to support its development has become a global consensus. Platform support, data drive, and inclusive sharing are its three primary characteristics. The digital economy is based on new-generation information technology, which gave birth to new business models and economic activities. Innovation in information technology improves the efficiency of resource allocation. As global industries undergo digital innovation, many countries witness the digital reform of financial institutions (Shaikh et al., 2017), the rise of the digital banking culture (Gruin and Knaack, 2020), and social currency digitization. Technological innovation fosters firms’ development and national competitiveness. As knowledge and technology-intensive entity, high-tech enterprises play a key role in promoting the transformation of scientific research results. The Yangtze River Delta region is the core of the urban agglomeration in the Yangtze River Economic Belt. The integrated development of the area helps promote the domestic economic cycle. Since a national strategy revolves around this region, financial technology talents, technology, capital, information, and other resources have been effectively integrated. The regional financial system has been continuously improved.

Technological innovation promotes scientific and technological research and corporate development activities, encouraging changes in the financial environment. The “14th Five-Year Plan” proposed that the national governance efficiency should be further improved. The Chinese state has vigorously promoted the development of inclusive digital finance. The development of big data, 5G, and artificial intelligence (AI) has enabled financial technology to support corporate operations. Digital financial inclusion gained momentum in the financial sector due to the Internet. It has expanded the coverage of financial services, improved financial risk control, and increased the availability of loans for small and medium-sized enterprises (SMEs) (Berger and Udell, 2006). It allowed greater inclusion into the financial system, and it is expected to support the financial service industry in the future. Existing research on digital financial inclusion mostly focuses on its macroeconomic aspects. Allen et al. (2016) found that financial inclusion expands employment opportunities and increases income levels. In addition, digital financial inclusion significantly affects innovation and entrepreneurship (Xie et al., 2018), household consumption (Yi and Zhou, 2018; Ding et al., 2019; Cheng and Gong, 2020), and industrial structure upgrades (Tang et al., 2019). Inclusive finance addresses the financing problems of disadvantaged groups in the market; hence, existing research on micro-enterprises primarily investigates SMEs’ financing constraints (Wan et al., 2020; Yu and Dou, 2020).

In the market economy, enterprises find it is difficult to meet their capital needs solely relying on internal financing. Firms often need to receive funds from other economic entities. Company executives often decide how companies raise funds. Different financing models will have a certain effect on organizational performance especially in the digital economy environment. External financing plays an essential role in corporate innovation (Fernandez, 2017; Shah et al., 2019; Wellalage and Fernandez, 2019). Savings may be converted into investment in daily production and operations.

Overall, the previous research agrees on the following aspects. (1) Substantial research on digital financial inclusion exists at the macro level. However, its impact on micro-enterprises innovation activities needs further discussion, especially considering the rapid development of financial technology. Whether inclusive finance promotes the realization of corporate innovation should be further investigated. (2) Most research on external financing addresses debt financing obtained from banks. In contrast, the comparative analysis of equity financing and debt financing is seldom conducted whether differences exist in how debt and equity financing influence enterprise innovation activities should be investigated. (3) With the digitalization of inclusive finance, various studies have qualitatively analyzed its development history and influence on people’s livelihood and consumption patterns. Quantitative analysis has primarily focused on macro-industry levels, addressing entrepreneurial activities and the financing environment. However, the relationship between digital financial inclusion and a company’s existing external financing and financing structure should be further explored.

This study focuses on whether the digital financial inclusion environment and external source financing significantly impact the innovation performance of high-tech enterprises. In this respect, the study’s results complement existing research on financial inclusion. Moreover, this study focuses on the Yangtze River Delta region, addressing enterprises in four provinces and cities of the Yangtze River Delta, further contributing to research in the field.

The study had structured in the following way: section “Introduction” provides the overview of the paper while briefly explaining the topic’s background. While section “Theoretical Framework and Research Hypotheses,” the literature review, investigates the research question backed by previous knowledge. Moreover, section “Materials and Methods” discusses the study methodology and results presented in section “Results.” Similarly, section “The Nature of Corporate Ownership” discusses corporate ownership, and section “Conclusion” concludes the findings by proposing implications and final wording on the study.

## Theoretical Framework and Research Hypotheses

### Digital Financial Inclusion and Innovation Performance

Global digital financial inclusion can be classified based on service forms and institutional changes. It has experienced an evolution of the type microfinance → inclusive finance → inclusive digital finance (Hu and Cheng, 2020). According to “External Control of Organizations” published by Salancik and Pfeffer (1978), the core hypothesis of resource dependence theory is organizations need to survive by acquiring resources in the environment. No organization is self-sufficient and must exchange with the environment. Organizations should be regarded as political actors rather than just work organizations that accomplish tasks. The resource dependence theory puts forward four important assumptions: (1) the most important thing for an organization is to care about survival; (2) in order to survive, the organization needs resources, and the organization itself usually cannot produce these resources; (3) the organization must be related to what it depends on. The factors in the environment interact, these factors usually include other organizations; (4) the survival of an organization is based on the ability to control its relationship with other organizations. It shows that the heterogeneous resources owned by the organization are the source of its competitive advantage and the determinants of performance differences between the organizations. The development of inclusive digital finance has evolved as a national strategy in China, becoming a driving force for supply-side structural reforms (Wu, 2019; Ajaz et al., 2020). Inclusive digital finance has improved firms’ external financial environment, optimizing financing conditions (Shen et al., 2010) and helping them solve practical development issues. Therefore, under the inclusive finance policy, high-tech enterprises receiving government subsidies and enjoying preferential tax policies increase the enterprise’s economic benefits and release a positive signal outside the organization, helping firms attract external financing (Liu et al., 2020) and improving their competitiveness. The development of digital financial inclusion has also made innovative products more accessible to traditional finance, effectively alleviating the problems of high financing costs and low efficiency in the traditional financial service industry (Berger and Udell, 2006; Allen et al., 2016). Innovation faces high upfront costs in technology-intensive high-tech enterprises, especially in the case of small and micro-enterprises, and substantial uncertainty (Sarfraz et al., 2020; Wan et al., 2020). Digital financial inclusion reduces the financial risks of early technology research and the development of enterprises. Enterprises use external financing and internal financing channels to protect their innovative activities. Using financial technology, digital financial inclusion reduces corporate financial services and labor costs through the Internet, alleviating corporate financing constraints (Yu and Dou, 2020), and attracting people who the modern financial service industry has long excluded. The financial system has improved the availability of financial services for SMEs and other underserved “long-tail” consumer groups (Cheng and Gong, 2020). It has enhanced the development vitality of the financial industry and provided enterprises with a rather innovative ecological environment (Hu et al., 2019). Hence, we propose the following hypothesis:

H1: Digital financial inclusion has a positive impact on the innovation performance of high-tech enterprises.

### Digital Financial Inclusion, External Financing, and Innovation Performance

External financing raises funds from outside the company, usually through equity financing or debt financing. Existing research suggests that external financing plays an essential role in corporate innovation (Ayyagari et al., 2011; Fernandez, 2017). Fernandez (2017) found that small innovative companies mainly rely on bank financing (Wellalage and Fernandez, 2019). Using corporate data from Eastern Europe and Central Asia, the study proposes that bank financing has a greater impact on corporate innovation; hence, innovation policy increases SMEs’ external financing opportunities. External financing provides stronger support to technology companies, which usually face high R&D risks. Gonzalez and James (2007) addressed United States-listed technology companies, showing that they are more likely to obtain bank loans, while the current income and cash flow aspects are less relevant. Egger and Keuschnigg (2017) and Neuhann and Saidi (2018) found that banks are willing to provide loans to production companies, even if they are risky.

Research on external financing considers two aspects: debt financing and equity financing. Bank loans represent debt financing. Commercial banks obtain interest income by providing short-term and long-term loans to enterprises. At the same time, firms receive a fair capital turnover. Equity financing implies the introduction of new shareholders to obtain financing without repayment of principal and interest and has a long-term nature. Both are fundamental channels for enterprises to obtain external funds. With the digital development of inclusive finance in China, corporate financing costs have gradually decreased at the technical level, and financing channels have continued to expand. The widespread promotion of inclusive digital finance has enabled inclusive finance to be applied to enterprise operations together with digital innovation (Shah et al., 2021). As a result, R&D risks can be compensated to some extent, and R&D enterprise innovation results can be promoted. Jorgenson’s neoclassical investment model pointed out that the government promotes the inclusiveness of finance through financial discounts and tax rebates, among others, to reduce the cost of capital for enterprises. The government implements an inclusive financial policy and provides incentives and subsidies to banks and other financial institutions to make it easier to approve loans to technology-based SMEs, stimulate R&D investment, and extend the inclusive financial policy to SMEs and the general public. Doing so, the government promotes mass entrepreneurship, further increases the availability of debt financing and equity financing for enterprises, and stimulates the vitality of enterprise innovation. When a company has sufficient external financing support, its technological research, and development capacity increase, promoting the digital innovation of the industry and the efficiency of the research and development output. The impact of digital financial inclusion on corporate innovation performance is inseparable from its interaction with external financing. Hence, we propose the following hypotheses:

H2a: The higher the level of corporate debt financing, the stronger the role of digital financial inclusion in promoting corporate innovation performance.
H2b: The higher the level of corporate equity financing, the stronger the role of digital financial inclusion in promoting corporate innovation performance.

**FIGURE 1:**
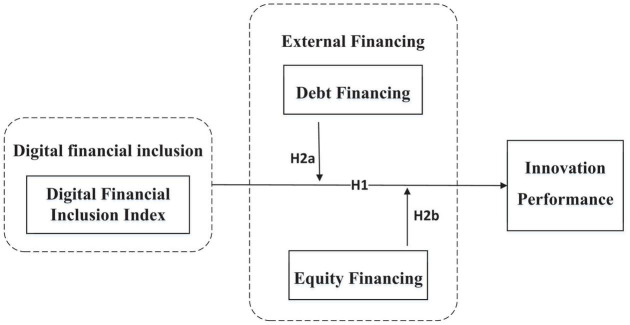
Theoretical model diagram.

The proposed theoretical model is shown in Figure 1.

## Materials and Methods

### Sample Selection and Data Sources

The data of this research comes from the China Stock Market & Accounting Research (CSMAR) database. CSMAR database is an economic and financial field developed by Shenzhen Sysma Data Technology Co., Ltd. After 21 years of continuous accumulation and improvement, the CSMAR database has covered 18 series including factor research, character characteristics, green economy, stocks, companies, overseas, information, funds, bonds, industries, economics, and commodity futures, including 160+ databases, more than 4,000 tables, 50,000 fields. In addition, based on the “Administrative Measures for the Recognition of High-tech Enterprises” promulgated in 2012, this study examines the panel data obtained from 112 high-tech companies in the Yangtze River Delta region, Shanghai, Zhejiang, Jiangsu, and Anhui. The province and city data cover the period between 2011 and 2020 and are obtained from “The Peking University Digital Financial Inclusion Index of China (PKU-DFIIC)” released by Peking University Digital Finance Research Center and Ant Financial Group. The initial sampling criteria are as follows:

(1) Companies with special treatments such as ST, ^∗^ST, and delisting are excluded;
(2) To ensure data integrity, companies with incomplete data disclosure are eliminated;
(3) Key indicators with missing observations are excluded.

Finally, 112 high-tech enterprises’ data in the four provinces are selected. The data used for analysis are mainly obtained from the CSMAR database, wind database, and Juchao Information Network, and processed using Stata15.0 statistical software. And for companies lacking patent application data information, we use the information provided by the National Intellectual Property Office (CNIPA) to manually supplement. Furthermore, to reduce the influence of outliers, this study minorize the upper and lower 1% quantiles on all continuous variables.

### Variable Selection and Measurement

The measurement and identification of the variables used for analysis are shown in Table 1. The independent variables in this study are digital financial inclusion and external financing. Digital financial inclusion is proxied by China’s Digital Financial Inclusion Index (DFIIC) (*fin*) (Guo et al., 2020), which represents the development status of digital financial inclusion in the region. The larger the index, the higher the level of digital financial inclusion in the region. At the city level, the China Digital Financial Inclusive Development Index developed by the Peking University Digital Finance Research Center and Ant Financial Services Group is employed to describe the inclusiveness and development of digital finance at the regional level in China. The index is comprehensively measured using the coverage breadth, usage depth, and digitization level. As mentioned above, external financing comprises debt financing and equity financing. In line with the literature, debt financing is expressed by the proportion of the total short-term and long-term loans in the total assets of the current period. Equity financing is proxied by current changes in the company’s equity and capital reserves as a proportion of total assets.

**TABLE 1:** Variable names and identification.

Types		Identification	Name	Measurement method
Dependent variable		*innov*	Innovation performance	Number of patent applications (inventions, utility models, and designs)
Independent variables	Digital financial inclusion	*fin*	Digital financial inclusion index	Jointly prepared by the Digital Finance Research Center of Peking University and Ant Financial Group
		*co*	Coverage breadth	
		*de*	Usage depth	
	External financing	*loan*	Debt financing	Debt financing = (short-term loans + long-term loans) / total assets
		*sto*	Equity financing	Equity financing = (current changes in the equity + changes in capital reserve) / total assets
Control variables		*fixas*	Fixed asset ratio	Fixed asset ratio = net fixed assets / total assets
		*intanas*	Intangible assets ratio	Intangible assets ratio = net intangible assets / total assets
		*lev*	Financial leverage	Financial leverage = total liability / total assets
		*tanas*	Tangible assets ratio	Tangible assets ratio = (total assets − net intangible assets − net value of goodwill) / total assets
		*age*	The age of the enterprise	The number of years since the company was founded
		*size*	The size of the enterprise	Natural logarithm of total assets
		Year	Time effect	Virtual variable
		Industry	Industry effect	Virtual variable, set up according to the 2012 classification standards of the China Securities Regulatory Commission

The dependent variable in this study is innovation performance (*innov*). Its measurement is in line with Xie and Zuo (2013), Guoqing et al. (2014), and Meuleman and De Maeseneire (2012). The number of patent applications of a company in the current year is used as a measure of innovation performance, including the sum of inventions, utility models, and designs. Missing data are manually supplemented utilizing the company’s annual reports and data from the National Intellectual Property Office (CNIPA). According to previous studies, the age and size of a company significantly impact their innovation performance. High-tech companies are also affected by the external influences of company growth and industry differences. Therefore, to reduce the interference of other variables on the results, in line with Li and Zhao (2016) and Liu et al. (2019), we control the age of the enterprise (*age*), its size (*size*), the fixed asset ratio (*fixas*), intangible assets ratio (*intanas*), financial leverage (*lev*), and tangible assets ratio (*tanas*). The age of an enterprise is the number of years since its establishment. Its size is the natural logarithm of its total assets. The ratio of fixed assets is expressed as the ratio of net fixed assets to total assets. The same is true for intangible assets. Financial leverage is expressed as the ratio of total liabilities to total assets. Tangible assets are expressed as the proportion of total assets after deducting intangible assets and the net value of goodwill in total assets. This study controls the year and industry effects. The dummy for the value of the enterprise in the year is equal to 1, and 0 otherwise. The industry effect is also proxied by a dummy variable set according to the 2012 classification standard of the China Securities Regulatory Commission.

### Regression Model

This study develops the following three regression models to test the proposed research hypotheses:


(1) $$I\,n\,n\,o\,v_{i,t}=\alpha_{0}+\beta_{0}\,f\,i\,n_{i,t}+\sum_{i,t}c\,o\,n\,t\,r\,o\,l+\mu_{0};$$

(2) $$I\,n\,n\,o\,v_{i,t}=\alpha_{1}+\beta_{1}\,f\,i\,n_{i,t}+\Theta_{1}\,\left(f\,i\,n_{i,t}\times l\,o\,a\,n_{i,t}\right)+\lambda_{1}\,l\,o\,a\,n_{i,t}+\sum_{i,t}c\,o\,n\,t\,r\,o\,l+\mu_{1};$$

(3) $$I\,n\,n\,o\,v_{i,t}=\alpha_{2}+\beta_{2}\,f\,i\,n_{i,t}+\Theta_{2}\,\left(f\,i\,n_{i,t}\times s\,t\,o_{i,t}\right)+\lambda_{2}\,s\,t\,o_{i,t}+\sum_{i,t}c\,o\,n\,t\,r\,o\,l+\mu_{2}.$$

*Innov*_*i*,*t*_ represents the innovation performance of company *i* in year *t*. *Fin*_*i*,*t*_ is the DFIIC of the province where the company is located in year *t*, *loan*_*i*,*t*_ represents its debt financing level, *sto*_*i*,*t*_ is the equity financing level, and β_0_ is the total effect of digital financial inclusion on innovation performance. In addition, *fin*_*i*,*t*_ × *loan*_*i*,*t*_ is the interaction between digital financial inclusion and debt financing, *fin*_*i*,*t*_ × *sto*_*i*,*t*_ represents the interaction between digital financial inclusion and equity financing, and ∑_*i*,*t*_ *control* is a vector of control variables, including enterprise age, size, fixed asset ratio, intangible asset ratio, and financial leverage, among others, while α is a constant, and μ is a random disturbance term.

## Results

### Descriptive Statistics and Correlation Analysis

Tables 2, 3 report the descriptive statistics and correlation analysis of the variables used for the analysis. A significant correlation is observed between the main variables. A large difference exists between the minimum (1) and maximum (338) enterprises’ innovation performance, with an average value of 22.84. The sample firms have a large gap in innovation performance, and their overall innovation performance is low. The average value of corporate equity financing is high, while the average debt financing is low. The median value of the three indicators of digital financial inclusion is slightly higher than the simple average between the corresponding maximum and minimum values, indicating that there is no significant difference in the degree of promotion and development of digital financial inclusion in the four provinces and cities of the Yangtze River Delta. From the perspective of the enterprise scale, the median value is 19.90, a relatively high level compared to the minimum (11.00) and the maximum value (24.36). Substantial differences are observed in the sizes of sample enterprises.

**TABLE 2:** Descriptive statistics.

Variable	Obs	Mean	SD	Min	Max
*innov*	724	22.84	51.02	1	338
*fin*	724	26.24	7.71	7.74	37.77
*co*	724	24.13	7.18	6.67	34.63
*de*	724	28.10	8.15	8.62	40.04
*sto*	724	15.00	7.014	0	22.74
*loan*	724	6.473	3.744	0	9.62
*fixas*	724	104.42	106.24	0.47	671.62
*intanas*	724	25.93	26.79	0	197.23
*tanas*	724	89.70	137.94	35.85	100
*lev*	724	1.04	2.80	−25.48	69.27
*age*	724	16.95	5.79	2	33
*size*	724	19.90	0.99	11.00	24.36

**TABLE 3:** Digital financial inclusion and innovation performance: primary results.

	(1)	(2)	(3)
	
Variables	Innov	Innov	Innov
*fin*	0.446*	1.505*	1.523*
	(2.072)	(2.411)	(2.252)
*loan*		−0.042*	
		(−1.820)	
*fin* × *loan*		8.416***	
		(2.766)	
*sto*			−0.056
			(0.363)
*fin* × *sto*			0.014
			(0.012)
**Control variables**			
*fixas*	−0.033*	−0.034*	−0.028*
	(−2.331)	(−2.439)	(−1.992)
*intanas*	0.144***	0.132*	0.115*
	(2.631)	(2.435)	(2.101)
*tanas*	0.033***	0.032***	0.030***
	(2.901)	(2.816)	(2.725)
*lev*	1.931	1.991	1.018
	(0.987)	(1.025)	(0.531)
*age*	−0.318	−0.272	−0.261
	(−1.266)	(−1.088)	(−1.054)
*size*	12.397***	12.249***	14.493***
	(4.269)	(4.240)	(4.837)
Constant	−264.131***	−528.792***	−895.925***
	(−7.197)	(−5.233)	(−7.602)
Year	Control	Control	Control
ID	Control	Control	Control
Observations	724	693	681
*R*-squared	0.151	0.166	0.158

Robust standard errors in parentheses, ***p < 0.01, **p < 0.05, *p < 0.1.

### Empirical Results

This study uses panel fixed effects to verify the relationship between digital financial inclusion and innovation performance (Models 1–3 in Table 3). The results of Model 1 show that after controlling for the related control variables and the enterprises and time fixed effects, digital financial inclusion has a positive and significant relationship with the innovation performance of high-tech enterprises (*p* < 0.1). The result is consistent with previous research results and theoretical analysis. The development of digital financial inclusion optimizes the financial environment. Technological innovation improves the convenience of corporate financing, promoting corporate R&D and improving high-tech enterprises’ innovation performance. Hence, H1 is verified.

To examine the influence of digital financial inclusion on corporate innovation in different external financing scenarios, the effects of the interactions between digital financial inclusion, debt financing, and equity financing are considered.

The results of Model 2 indicate that the coefficient on the interaction between digital financial inclusion and debt financing is positive and significant at the 1% level. With an increase in the level of corporate debt financing, the role of digital financial inclusion in promoting corporate innovation performance also increases. The results of Model 3 show that the coefficient on the interaction between digital financial inclusion and debt financing is not statistically significant. Compared with equity financing, the higher the level of debt financing, the stronger the role of digital financial inclusion in promoting innovation performance. Hence, H2a is verified, while H2b is not. A possible explanation is that the approval procedures for external equity financing, such as additional issuance or allotment of Chinese listed companies, are complicated and demanding. Chinese firms cannot stably rely on equity financing as a source of funds. Hence, companies tend to focus on debt financing.

To further verify the relationship between digital financial inclusion, external financing, and corporate innovation performance, this study used the measurements proposed by Liao et al. (2020). In line with the trend of the China Digital Financial Inclusive Development Index, this approach acknowledges that the Digital Support Service Level Index has largely fluctuated since 2011. The proposed measures use the two first-level indexes of the coverage breadth (*co*) and usage depth (*de*) of digital financial inclusion to assess the development and impact of digital financial inclusion. Table 4 shows the results for the interaction between the coverage breadth and usage depth of digital financial inclusion and external financing.

**TABLE 4:** Coverage breadth and depth of use of digital financial inclusion and innovation performance.

	*X*: coverage breadth			*X*: usage depth		
Variables	(1)	(2)	(3)	(1)	(2)	(3)
*X*	0.022***	0.047***	0.082	2.644	2.13	−1.8
	(4.629)	(3.240)	(1.561)	(2.105)	(2.13)	(1.6)
*loan*		−0.001*			−3.65	
		(−1.845)			(3.85)	
*X* × *loan*		0.064*			−2.405	
		(1.197)			(3.20)	
*sto*			−0.166***			−0.9
			(−2.914)			(4.5)
*X* × *sto*			−2.171			−3.4
			(−0.937)			(3.1)
Control variables:	Control	Control	Control	Control	Control	Control
Year	Control	Control	Control	Control	Control	Control
ID	Control	Control	Control	Control	Control	Control
Constant	0.480	−2.171	0.156	5.500	−3.241	0.375
	(0.584)	(−0.937)	(0.188)	(−2.168)	(−0.995)	(0.846)
Observations	724	693	681	724	693	681
*R*-squared	0.413	0.534	0.537	0.184	0.123	0.129

Robust standard errors in parentheses, ***p < 0.01, **p < 0.05, *p < 0.1.

The results for the coverage breadth of digital financial inclusion show that when external financing is not considered, the breadth of coverage has a significant positive relationship with innovation performance (*p* < 0.01). The coefficient on the interaction between coverage breadth and debt financing is positive and significant at 10% level. With an increase in the level of corporate debt financing, the coverage of digital financial inclusion increases the promotion of corporate innovation performance, in line with previous results on the comprehensive DFIIC.

The results for the usage depth of digital financial inclusion indicate that when external financing is not considered, the positive relationship between the depth of use and innovation performance is not significant. The coefficient on the interaction with external financing is not statistically significant. This result may be due to the fact that although digital financial inclusion has been widely promoted in China, the degree of digitalization of enterprises is still low, and digitalization has not yet penetrated the production and operation stages. The impact of the usage depth of inclusive finance on corporate innovation remains to be verified over time.

## The Nature of Corporate Ownership

In a socialist system with Chinese characteristics, the relationship between digital financial inclusion, external financing, and corporate innovation may vary due to differences in corporate ownership. Therefore, this study divides the sample into state-owned enterprises (SOEs) and non-SOEs and studies the effect of corporate ownership in the proposed theoretical model.

State-owned enterprises are mainly controlled by the government and are heavily supported by national policies. Hence, the R&D investment of state-owned high-tech enterprises is likely to be affected differently by economic policies. In addition, external financing is the main channel of R&D financing for SOEs. Since equity financing costs are relatively low and enjoy tax incentives, risk-averse SOEs are more inclined to equity financing. The results in Table 5 indicates that for SOEs, the comprehensive DFIIC, coverage breadth, and usage depth all play a significant role in promoting innovation performance. The coefficient on the interaction between equity financing and the three indicators are positive and significant, while the effect of the interaction between debt financing and the coverage breadth of digital financial inclusion is not significant. It may be because for SOEs, the effect of equity financing is slightly higher than that of debt financing. The latter has not yet reached optimality in promoting innovation performance, and debt financing policies targeting SOEs still need improvement.

**TABLE 5:** State-owned enterprises.

	(1)	(1)	(1)	(2)	(2)	(2)	(3)	(3)	(3)
	
Variables	Innov	Innov	Innov	Innov	Innov	Innov	Innov	Innov	Innov
*fin*	11.95***			9.32***			7.35***		
	(0.60)			(1.035)			(0.354)		
*co*		15.45***			14.62***			10.56***	
		(1.033)			(1.93)			(0.847)	
*de*			7.11***			2.88***			1.77*
			(0.392)			(0.445)			(0.654)
*loan*				12.57**	−4.86	23.91***			
				(3.29)	(4.62)	(2.08)			
*fin* × *loan*				0.19*					
				(0.068)					
*co* × *loan*					−0.03				
					(0.112)				
*de* × *loan*						0.42***			
						(0.03)			
*sto*							−9.11**	9.270***	10.79**
							(1.86)	(1.067)	(2.89)
*fin* × *sto*							0.32**		
							(0.05)		
*co* × *sto*								0.339***	
								(0.025)	
*de* × *sto*									0.38**
									(0.07)
Control variables:	Control	Control	Control	Control	Control	Control	Control	Control	Control
Constant	327.0**	396.75**	205.28**	272.30**	350.46*	154.33***	457.19**	547.62**	293.25***
	(67.99)	(95.82)	(39.27)	(68.31)	(110.79)	(22.66)	(97.20)	(113.66)	(46.74)
Year	Control	Control	Control	Control	Control	Control	Control	Control	Control
ID	Control	Control	Control	Control	Control	Control	Control	Control	Control
N	216	216	216	216	216	216	216	216	216
*R*-squared	0.67	0.716	0.559	0.688	0.727	0.601	0.697	0.743	0.605

Robust standard errors in parentheses, ***p < 0.01, **p < 0.05, *p < 0.1.

In contrast, non-state-owned firms mainly depend on the market for financing and face higher risks. Therefore, they typically have stronger risk tolerance than SOEs and focus on the economic utility of external funds, followed by the level of external financing costs. Therefore, the external financing of non-SOEs usually relies on debt financing methods, such as bank loans, for supporting R&D. The results in Table 6 shows that for non-SOEs, the effect of the interaction between external financing and the comprehensive index of digital financial inclusion is consistent with the results for SOEs. In terms of the coverage breadth and usage depth, the coefficients on the interaction between debt financing and the three indicators are all positive and significant, while the interaction effects of equity financing and the usage depth of digital financial inclusion are not significant. In the external financing of non-SOEs, the effect of debt financing is slightly higher than that of equity financing, suggesting that non-SOEs’ equity financing has not yet reached optimality in promoting innovation performance.

**TABLE 6:** Non-state-owned enterprises.

	(1)	(1)	(1)	(2)	(2)	(2)	(3)	(3)	(3)
	
Variables	Innov	Innov	Innov	Innov	Innov	Innov	Innov	Innov	Innov
*fin*	0.95***			0.340*			0.331		
	(0.28)			(0.1718)			(0.349)		
*co*		1.074***			0.419*			0.379	
		(0.287)			(0.204)			(0.345)	
*de*			0.755**			0.202			0.457
			(0.324)			(0.176)			(0.36)
*loan*				5.27***	−5.10***	−5.17**			
				(1.674)	(1.562)	(1.91)			
*fin* × *loan*				0.112***					
				(0.02)					
*co* × *loan*					0.114***				
					(0.021)				
*de* × *loan*						0.102***			
						(0.025)			
*sto*							−0.299	−0.357	0.194
							(0.468)	(0.462)	(0.466)
*fin* × *sto*							0.039**		
							(0.017)		
*co* × *sto*								0.043**	
								(0.019)	
*de* × *sto*									0.019
									(0.014)
Control variables:	Control	Control	Control	Control	Control	Control	Control	Control	Control
Constant	67.37**	70.03***	62.19***	76.27**	−79.11**	70.18**	66.96**	68.61***	68.53**
	(19.84)	(20.31)	(20.26)	(31.47)	(32.94)	(31.34)	(23.209)	(22.529)	(25.69)
Year	Control	Control	Control	Control	Control	Control	Control	Control	Control
ID	Control	Control	Control	Control	Control	Control	Control	Control	Control
*N*	508	508	508	508	508	508	508	508	508
*R*-squared	0.18	0.182	0.171	0.2023	0.20378	0.1925	0.1974	0.198	0.189

Robust standard errors in parentheses, ***p < 0.01, **p < 0.05, *p < 0.1.

## Conclusion

This study examines the relationship between digital financial inclusion and corporate innovation performance, as well as the relationship between external financing, financial inclusion, and corporate innovation, in the context of the digital economy. By introducing the inclusive digital finance coverage breadth and usage depth, we construct a research framework that links inclusive digital finance, external financing, and the innovation performance of high-tech enterprises. The regional data of three provinces and one city in the Yangtze River Delta are employed to study the interaction between digital financial inclusion and external financing. On this basis, we study the in-depth influence of the nature of corporate ownership.

The theoretical contribution of this research is twofold. First, by verifying the impact of the interaction between digital financial inclusion and external financing, this study enriches existing knowledge of digital financial inclusion and the path mechanism between innovation and innovation performance. Second, the study combines macro- and micro-level approaches. It addresses regional external financing channels, the development of digital financial inclusion, and micro-enterprise practices, clarifying the different mechanisms of action at play in the relationship between digital financial inclusion, external financing, and corporate innovation performance.

The research in this article also has certain shortcomings. The research scope belongs to regional research. In the future, the sample size can be expanded and further studies can be carried out on specific corporate practices.

### Policy Recommendations

This study focuses on high-tech enterprises in the Yangtze River Delta, China. The integration of regional financial technology talents, technology, capital, and other resources is relatively complete in this region; hence, the problems reflected in its economic data are forward-looking and a reference for future research. This study proposes the following policy recommendations.

(1) Further strengthen the usage depth of digital financial inclusion and popularize the digital degree of financial inclusion at the technical level. In promoting innovation performance, the impact of the interaction between the usage depth of digital financial inclusion and external financing is not significant. Although digital financial inclusion has been widely promoted in the country, its degree of digitalization has not yet penetrated into the daily operations of enterprises in the Yangtze River Delta region.
(2) Further improving incentives for enterprises’ external financing policies. The external financing strategy of high-tech enterprises should further refine specific measures based on the characteristics of corporate ownership. Equity financing can better promote the usage depth of digital financial inclusion in SOEs, while debt financing can promote the coverage breadth of non-SOEs. Therefore, support for high-tech companies should be further increased at the level of financing policy formulation.
(3) Technological innovation is the driving force behind the development of enterprises. R&D based on big data, AI, cloud computing, and other technologies should be further strengthened, and enterprises should be encouraged to use big data for technological upgrades and innovation. While strengthening technological innovation, it is necessary to nurture the long-term development of enterprises through the digital transformation of inclusive finance.

## Data Availability Statement

Publicly available datasets were analyzed in this study. This data can be found here: The Peking University Digital Financial Inclusion Index of China (PKU-DFIIC), available at https://idf.pku.edu.cn/zsbz/515313.htm

## Author Contributions

Both authors listed have made a substantial, direct, and intellectual contribution to the work, and approved it for publication.

## Conflict of Interest

The authors declare that the research was conducted in the absence of any commercial or financial relationships that could be construed as a potential conflict of interest.

## Publisher’s Note

All claims expressed in this article are solely those of the authors and do not necessarily represent those of their affiliated organizations, or those of the publisher, the editors and the reviewers. Any product that may be evaluated in this article, or claim that may be made by its manufacturer, is not guaranteed or endorsed by the publisher.

## References

[B1] Ajaz A. Shenbei Z. Sarfraz M. (2020). Delineating the influence of boardroom gender diversity on corporate social responsibility, financial performance, and reputation. *Logforum* 16 61–74. 10.17270/J.LOG.2020.376

[B2] Allen F. Demirguc-Kunt A. Klapper L. Peria M. S. M. (2016). The foundations of financial inclusion: understanding ownership and use of formal accounts. *J. Financ. Int.* 27 1–30. 10.1016/j.jfi.2015.12.003

[B3] Ayyagari M. Demirgüç-Kunt A. Maksimovic V. (2011). Firm innovation in emerging markets: the role of finance, governance, and competition. *J. Financ. Quant. Anal.* 46 1545–1580. 10.1017/S0022109011000378

[B4] Berger A. N. Udell G. F. (2006). A more complete conceptual framework for SME finance. *J. Bank. Finance* 30 2945–2966. 10.1016/j.jbankfin.2006.05.008

[B5] Cheng X. Gong Q. (2020). How does digital financial inclusion affect the development of the real economy —Based on the analysis of the system GMM model and the intermediary effect test. *J. Hunan Univ. (Soc. Sci. Edn.)* 34 59–67.

[B6] Ding R. Liu R. Zhang Q. (2019). Research on the impact and mechanism of digital inclusive finance on the development of service industry—an empirical analysis based on inter-provincial panel data. *Finance Econ*. 07, 4–10.

[B7] Egger P. H. Keuschnigg C. (2017). Access to credit and comparative advantage. *Can. J. Econ.* 50 481–505. 10.1111/caje.12266

[B8] Fernandez V. (2017). The finance of innovation in Latin America. *Int. Rev. Financ. Anal.* 53 37–47. 10.1016/j.irfa.2017.08.008

[B9] Gonzalez L. James C. (2007). Banks and bubbles: how good are bankers at spotting winners? *J. Financ. Econ.* 86 40–70. 10.1016/j.jfineco.2006.07.004

[B10] Gruin J. Knaack P. (2020). Not just another shadow bank: Chinese authoritarian capitalism and the ‘developmental’promise of digital financial innovation. *New Polit. Econ.* 25 370–387. 10.1080/13563467.2018.1562437

[B11] Guo F. Wang J. Wang F. Kong T. Zhang X. Cheng Z. (2020). Measuring China’s digital financial inclusion: index compilation and spatial characteristics. *China Econ. Q.* 19 1401–1418.

[B12] Guoqing L. Zhou W. Chunyu Z. (2014). Research on the performance of subsidizing innovation for Chinese strategic emerging industry. *Econ. Res. J.* 7 44–55.

[B13] Hu B. Cheng X. (2020). Financial technology, digital financial inclusion and national financial competitiveness. *J. Wuhan Univ. (Philos. Soc. Sci. Edn.)* 73 130–141.

[B14] Hu J. Jiang H. Holmes M. (2019). Government subsidies and corporate investment efficiency: evidence from China. *Emerg. Mark. Rev.* 41:100658. 10.1016/S0140-6736(19)30841-4 31030984 PMC6548764

[B15] Li C. Zhao X. (2016). Research on the effect of my country’s high-tech enterprises’ fiscal and tax incentives on R&D investment. *Tax. Res.* 2 105–109.

[B16] Liao J. Hu Y. Xiang H. (2020). Does the development of digital financial inclusion ease corporate financing constraints? —Based on the moderating effect of corporate social responsibility. *J. Yunnan Univ. Finance Econ.* 36 73–87.

[B17] Liu X. Kong X. Liu C. Wang Q. (2019). Do government subsidies promote independent innovation of enterprises? *J. Cap. Univ. Econ. Bus.* 21 85–93. 10.3390/ijerph16173084 31450652 PMC6747305

[B18] Liu X. Song H. Fan L. (2020). The signal transmission effect of government subsidies and enterprise innovation on investors’ investment decisions. *Sci. Technol. Progr. Policy* 37 26–33.

[B19] Meuleman M. De Maeseneire W. (2012). Do R&D subsidies affect SMEs’ access to external financing? *Res. Policy* 41 580–591. 10.1016/j.respol.2012.01.001

[B20] Neuhann D. Saidi F. (2018). Do universal banks finance riskier but more productive firms? *J. Financ. Econ.* 128 66–85. 10.1016/j.jfineco.2018.01.011

[B21] Salancik G. R. Pfeffer J. (1978). A social information processing approach to job attitudes and task design. *Adm. Sci. Q*. 23, 224–253. 10.2307/2392563 10307892

[B22] Sarfraz M. Shah S. G. M. Ivascu L. Qureshi M. A. A. (2020). Explicating the impact of hierarchical CEO succession on small-medium enterprises’ performance and cash holdings. *Int. J. Finance Econ.* 10.1002/ijfe.2289

[B23] Shah S. G. M. Sarfraz M. Ivascu L. (2021). Assessing the interrelationship corporate environmental responsibility, innovative strategies, cognitive and hierarchical CEO: a stakeholder theory perspective. *Corp. Soc. Responsib. Environ. Manag.* 28 457–473. 10.1002/csr.2061

[B24] Shah S. G. M. Sarfraz M. Fareed Z. Rehman M. A. Maqbool A. Qureshi M. A. A. (2019). Whether CEO succession via hierarchical jumps is detrimental or blessing in disguise? Evidence from Chinese listed firms. *Zagreb Int. Rev. Econ. Bus.* 22 23–41. 10.2478/zireb-2019-0018

[B25] Shaikh A. A. Glavee-Geo R. Karjaluoto H. (2017). Exploring the nexus between financial sector reforms and the emergence of digital banking culture–Evidences from a developing country. *Res. Int. Bus. Finance* 42 1030–1039. 10.1016/j.ribaf.2017.07.039

[B26] Shen H. Kou H. Zhang C. (2010). An empirical study of financial development, financing constraints and corporate investment. *China Industr. Econ.* 6 55–64. 10.1371/journal.pone.0247549 33735187 PMC7971474

[B27] Tang W. Li S. Tao Y. (2019). The development of digital inclusive finance and the upgrading of industrial structure—empirical evidence from 283 cities. *J. Guangdong Univ. Finance Econ*. 34, 35–49.

[B28] Wan J. Zhou Q. Xiao Y. (2020). Digital finance, financing constraints and enterprise innovation. *Econ. Rev.* 1 71–83.

[B29] Wellalage N. H. Fernandez V. (2019). Innovation and SME finance: evidence from developing countries. *Int. Rev. Financ. Anal.* 66:101370. 10.1016/j.irfa.2019.06.009

[B30] Wu S. (2019). Risk issues, regulatory challenges and development suggestions of digital financial inclusion. *Technoecon. Manag. Res.* 1 66–69.

[B31] Xie X. Zuo L. (2013). The characteristics of enterprise collaborative innovation network and innovation performance: a study on the mediating effect based on knowledge absorptive capacity. *Nankai Manag. Rev*. 16, 47–56.

[B32] Xie X. She Y. Zhang H. Guo F. (2018). Can digital financial inclusion promote entrepreneurship? —Evidence from China. *Economics (Quarterly)* 4 1557–1580.

[B33] Yi X. Zhou L. (2018). Is the development of digital financial inclusion significantly affecting household consumption—micro evidence from Chinese households. *Financial Res*. 11, 47–67.

[B34] Yu P. Dou J. (2020). Does the development of digital financial inclusion ease the financing constraints of SMEs? *Finance Account. Month.* 03 140–146.

